# Hereditary ovarian cancer risk reduction: a retrospective evaluation of patient perspectives and service provision at a regional hereditary gynaecologic cancer clinic 2006–2016

**DOI:** 10.1186/s12905-022-01844-5

**Published:** 2022-06-29

**Authors:** Lauren Adolph, Ashley Warias, Jocelyn Stairs, Kelly Collins-McNeil, Lynette Penney, Katharina Kieser

**Affiliations:** 1 Department of Obstetrics and Gynaecology, Dalhousie University, Halifax, NS Canada; 2 Maritime Medical Genetics, IWK Health Centre, Halifax, NS Canada; 3 Division of Medical Genetics, IWK Health Centre, Halifax, NS Canada; 4 Division of Gynaecologic Oncology, Dalhousie University, Dickson Building - Room 5007, 5820 University Avenue, Halifax, NS B3H 1V7 Canada

**Keywords:** Hereditary breast and ovarian cancer, Risk-reduction, Genetic counselling, Patients’ perspective

## Abstract

**Background:**

Germline pathogenic variants in *BRCA1/2* have been established in hereditary breast and ovarian cancer (HBOC) syndrome and result in significantly elevated lifetime risk of ovarian cancer. Risk reduction interventions are presently the only effective means of improving survival and specialized counselling clinics have been established as an effective means of aiding this population in navigating complex decisions surrounding these interventions. This study sought to evaluate patient perceptions of a specialized counselling clinic for patients with HBOC Syndrome and referral patterns to this clinic.

**Methods:**

We completed a retrospective review of 200 patients with HBOC in Nova Scotia, Canada seen through Maritime Medical Genetics Services between 2006 and 2016. Data were collected on referral pattern to the Hereditary Gynaecologic Risk Reduction Clinic (HGRRC), demographics, health history, and uptake of risk-reducing interventions. Participants were invited to complete a questionnaire about their experience.

**Results:**

156/200(78%) women were referred to HGRCC and 135/156 (85.9%) of those referred attended their appointment. 124/200 (62%) were over age 40 at the time of testing. The mean time from referral to HGRCC appointment was 134.68 days (SD 85.78). 85/135 (63%) underwent risk-reducing bilateral salpingo-oophorectomy following their HGRCC appointment. The questionnaire was completed by 94/188 (50.3%) women. Most participants found information received from genetics clinics (81/94; 91%) and genetic counsellors (87/94; 95%) most helpful in making choices around risk-reduction strategies. 83/94 (88%) participants felt they had sufficient information to make an informed decision.

**Conclusion:**

The majority of women with HBOC in Nova Scotia during the study period were referred to and counselled through HGRRC. Genetic counselling was found most valuable in risk-reduction decision making, which highlights the importance of a multidisciplinary team. Patients viewed this clinic as an effective care model to support informed choice about risk-reducing intervention.

**Supplementary Information:**

The online version contains supplementary material available at 10.1186/s12905-022-01844-5.

## Background

Germline pathogenic variants in *BRCA1/2* have been established in hereditary breast and ovarian cancer (HBOC) syndrome. Carriers of this pathogenic variant have an elevated lifetime risk of ovarian cancer of approximately 16–59% depending on the variant [1]. At present, there is no screening test for ovarian cancer shown to improve overall survival [2, 3]. Therefore, risk-reduction interventions are the only effective means of decreasing ovarian cancer deaths in this population. Options for risk reduction include the oral contraceptive pill (OCP), which confers a 50% reduction in risk [4], and risk-reducing bilateral salpingo-oophorectomy, which confers a 90% reduction [5–8]. It is recommended that women with *BRCA1/2* pathogenic variants receive counselling about their risk and are offered appropriate preventative strategies to mitigate the profound potential implications on life expectancy [9–11].

While risk-reducing BSO markedly reduces the risk of ovarian cancer, the personal decision to proceed requires considerations of future fertility; potential implications on bone, cardiac, and sexual health following surgical menopause; and the possibility of detecting an occult or overt malignancy. Specialized hereditary cancer counselling clinics have proven effective in disseminating essential counselling information and ensuring patients have access to timely risk-reduction strategies [12]. Prior studies of this clinical model demonstrated high appointment uptake among eligible women [13], and uptake of risk-reducing BSO ranged from 70 to 87% in women older than 40 years [13, 14]. However, few studies have examined patient perceptions of gynaecologic hereditary cancer counselling clinics.

Nova Scotia is a province of approximately 1 million people on the east coast of Canada. In 2021, there were 780 new cases of breast cancer and 75 new cases of ovarian cancer in Nova Scotia [15]. Care for patients with breast and ovarian cancer is provided by five academic breast surgeons in conjunction with community general surgeons, and four gynaecologic oncologists. Risk-reduction education has been offered for gynaecological malignancy in HBOC since 2005, and a multidisciplinary clinic, the Hereditary Gynaecologic Risk Reduction Clinic (HGRRC), was created in 2009. This model offers Nova Scotian women an appointment with Maritime Medical Genetics Service, which includes a team of medical geneticists and genetic counsellors who provide patients with their genetic diagnosis, an overview of their ovarian cancer risk, and options for risk-reduction. Patients then attend an appointment at HGRRC and are offered a group education session at the time of their appointment. This physician-led session includes education about *BRCA* ovarian cancer risk, risk-reduction strategies, and hormone replacement therapy in the context of *BRCA* carrier status. Patients are then seen individually by the clinical team for further personalized counselling and care planning.

The aim of this study was to examine the success of a specialized hereditary counselling clinic in Nova Scotia, Canada in the provision of risk-reduction information to women with pathogenic *BRCA1/2* variants. The first objective was to explore patient perceptions of the clinic. The second objective was to determine referral rates to HGRRC and assess factors associated with HGRRC attendance, including those influencing the uptake of risk-reducing BSO following HGRRC attendance.

## Methods

A retrospective chart review of all women diagnosed with a *BRCA1/2* pathogenic variant in Nova Scotia, Canada from January 1, 2006 to December 31, 2016 was conducted by obtaining data from two site-specific databases: the Maritime Medical Genetics database and the Tupper Gynaecologic Oncology database.

Women were included in the cohort if they had a breast cancer diagnosis that preceded their genetic diagnosis or developed breast cancer or epithelial ovarian cancer following confirmation of their *BRCA1/2* carrier status. Participant characteristics collected included year of birth, ethnicity (defined as patient self-reported heritage or region of origin), personal cancer history, family history of cancer, *BRCA* test result, date of referral to HGRRC, date of attendance at HGRRC, previous use of OCP, previous tubal ligation, previous salpingectomy or oophorectomy, previous risk-reducing surgery, diagnosis of STIC lesion(s) and diagnosis of ovarian cancer, including date and stage.

A questionnaire that assessed patients’ personal history of risk-reduction measures and sources of information that influenced this decision-making was administered using the RedCap 9.5.22 software interface [Additional file 1: Appendix] [16, 17]. Patients who had a confirmed *BRCA1/2* pathogenic variant diagnosed during the study period and who were still living at the time of data retrieval, were mailed a study letter of invitation. Four women who developed epithelial ovarian cancer following their genetic diagnosis were contacted separately by the principal investigator to assess interest in participating given the potentially sensitive nature of the questionnaire in this context. Responses from the questionnaire were submitted electronically, by hardcopy, or via telephone through a research assistant. Informed consent was implied by receipt of a completed questionnaire. Participants were given the option to be entered into a draw to win one of four gift cards (50 Canadian dollars) at the time of survey completion. Follow-up phone calls were made to non-respondents four-weeks from the time of initial mail out by a research assistant to encourage participation.

Patient and clinic referral characteristics and survey responses were analyzed using descriptive statistics. Continuous and categorical variables were compared using the Student’s t-test and chi-square test and Fisher’s exact test respectively. Statistical significance was defined as a two-tailed alpha level of 0.05. All analyses were performed using SAS STAT 14.1 software version 9.3 (SAS Institute, Cary, NC).

This study received research ethics board approval from the Nova Scotia Health Authority (Nova Scotia Health Authority REB #1022959).

## Results

Two-hundred women were diagnosed with a *BRCA1/2* pathogenic variant in Nova Scotia between January 1, 2006 and December 31, 2016. Testing was predictive for 75.5%. The mean age at genetic diagnosis was 45.1. 31.5% had a *BRCA1* and 68.5% a *BRCA2* pathogenic variant. Most participants (84%;166/200) were of Western European heritage. Overall, one third had objectively documented prior OCP use, 55.0% had undergone a BSO and 20.0% had undergone a risk-reducing unilateral or bilateral mastectomy. Twelve individuals were deceased at the time of data retrieval. Cohort characteristics are summarized in Table 1.

**Table 1:** Demographic characteristics of women diagnosed with a *BRCA1/2* pathogenic variant in Nova Scotia, Canada between January 1, 2006 and December 31, 2016

Characteristic	Patients with BRCA1/2 pathogenic variant (n = 200), n (%)
Heritage/region of origin (patient self report)	
Acadian	5 (2.5%)
Ashkenazi Jewish	5 (2.5%)
Western European	166 (83%)
Eastern European	^a^
First Nations	^a^
Nordic	5 (2.5%)
Other	5 (2.5%)
Unknown	10 (5%)
Personal history of breast cancer	
Yes	92 (46%)
No	108 (54%)
Personal history of ovarian cancer after genetic diagnosis	
Yes	^a^
No	196 (98%)
Personal history of other cancers	
Yes	11 (5.5%)
No	189 (94.5%)
Family history of breast cancer	
Yes	187 (82.5%)
No	13 (17.5%)
Family history of ovarian cancer	
Yes	83 (42.5%)
No	117 (58.5%)

^a^Censored for small sample size

Overall, 78.0% (156/200) women were referred to HGRRC and 86.5% (135/156) of women who were referred attended their appointment. Previous risk-reducing surgery, declining or missing an appointment, and extremes of age were the most common reasons for lack of referral. The mean time from referral to HGRRC appointment was 134.68 days (SD 85.78).

Of those women who attended their HGRRC appointment, 43.7% had previously been diagnosed with breast cancer and none had been diagnosed with epithelial ovarian cancer prior to attendance. Women who had previously had a prior hysterectomy or BSO for non-risk-reducing indications were less likely to attend. There were no other significant differences detected between women who attended HGRRC versus those that did not (Table 2). No women who attended HGRRC had undergone surgery for an indication of risk-reduction prior to their appointment.

**Table 2:** Factors Affecting Attendance at HGRRC among women diagnosed with a *BRCA1/2* pathogenic variant in Nova Scotia, Canada between January 1, 2006 and December 31, 2016

Factor	Attended (n = 135) n (%)	Declined (n = 65) n (%)	p-value
Age at time of genetic diagnosis, mean (SD)	44 (13)	46 (17)	0.78
Personal history of breast cancer	59 (44)	32 (50)	0.40
Personal history of ovarian cancer	3 (2)	1 (2)	1.00
Family history of breast cancer	126 (93)	60 (94)	0.91
Family history of ovarian cancer	62 (46)	21 (33)	0.08
Risk-reducing surgery prior to HGRRC attendance	0 (0)	8 (13)	< 0.001*
Hysterectomy for non-risk-reducing indication	6 (4)	12 (19)	0.001*
Tubal ligation for non-risk-reducing indication	20 (15)	4 (6)	0.10
BSO for non-risk-reducing indication	3 (2)	8 (13)	0.001*
Risk-reducing bilateral mastectomy	16 (12)	2 (3)	0.06

*Denotes statistical significance

Of the women who attended HGRRC and were eligible for risk-reducing surgery, 64.4% had a subsequent risk-reducing BSO and 18.6% (24/129) had a concomitant hysterectomy. Twenty-six women were 40 years of age or less at the time of their risk-reducing BSO (30.6%). Age at genetic diagnosis, personal history of breast cancer, and previous ever-use of an OCP were significantly associated with the decision to proceed with risk-reducing surgery (Table 3).

**Table 3:** Factors affecting the decision to pursue risk-reducing BSO among women diagnosed with a *BRCA1/2* pathogenic variant and who were eligible for risk-reducing BSO in Nova Scotia, Canada between January 1, 2006 and December 31, 2016

Factor	Pursued risk-reducing BSO (n = 85) n (%)	Did not pursue risk-reducing BSO (n = 47) n (%)	p-value
Age at time of genetic diagnosis, mean (SD)	46.8 (9.1)	39 (17)	< 0.001*
Personal history of breast cancer	46 (54)	12 (26)	0.002*
Personal history of ovarian cancer	1 (1)	0 (0)	1.00
Risk-reducing bilateral mastectomy	13 (15)	3 (6)	0.17
Family history of breast cancer	81 (95)	42 (89)	0.20
Family history of ovarian cancer	36 (42)	25 (53)	0.23
Previous use of OCP	27 (32)	26 (55)	0.008*
No prior use OCP	10 (12)	10 (21)	0.14

*Denotes statistical significance

Half of eligible women who were alive at the time of dissemination (94/188) completed the questionnaire. Respondents and non-respondents were similar in age (47 vs 43 years, p = 0.06) and breast cancer history (44/94; 46.8% vs 48/106; 45.3%, p = 0.83). Respondents were more likely than non-respondents to have a prior history of prior risk-reducing BSO (60/94; 63.8% vs 55/106; 51.9%, p = 0.04).

Most participants (74/94; 78.7%) indicated they were aware that they were offered an HGRCC appointment. Majority of participants (84/94; 89.4%) self-reported prior OCP use, with a mean duration of 10.27 years (SD7.97; range 0.25–36.0 years). However, most indicated the primary intent of OCP use was not for ovarian cancer risk reduction. Participants found information received from genetics clinic (81/94; 86.2%) and genetic counsellors (87/94; 92.6%) most helpful in making choices around risk-reduction strategies. Overall, 83/94 (88.3%) participants felt they had sufficient information to make an informed decision.

## Discussion

The decision to pursue surgical risk-reduction intervention is medically and emotionally complex and requires transparent, comprehensive, and timely counselling on potential benefits and risks. This study demonstrates that a patient education program delivered through a multidisciplinary counselling clinic had high uptake by eligible patients and that patients diagnosed with a *BRCA1/2* pathogenic variant who responded to the questionnaire were satisfied with the level of information received and felt well-informed to make decisions about ovarian cancer risk-reduction.

In this cohort, 78% of eligible women were referred and 86.5% of these attended the HGRRC clinic. This is concordant with existing studies that utilize a similar approach to risk-reduction counselling [13, 14]. Patients’ interest and willingness to attend the clinic highlights the perceived utility of this care model. Further, prior studies examining uptake of risk-reducing BSO demonstrate uptake ranging from 12 to 83% [18–20]. The rate of risk-reducing BSO uptake in our cohort was consistent with this. Other studies have identified older age, having had children, personal history of breast cancer, family history of ovarian cancer, or risk-reducing mastectomy as positive factors influencing a patient’s decision to undertake risk-reducing BSO [18, 19]. In our study population, age at genetic diagnosis and prior history of breast cancer were positive predictive factors for risk-reducing BSO uptake. This suggests that there may be an opportunity to improve education highlighting risk-reducing BSO as a risk reduction option to older patients and those without a personal history of breast cancer to ensure all patients at risk are engaged in informed decision-making.

While ovarian cancers in *BRCA1/2* carriers generally develop in the 4th or 5th decades of life, evidence suggests that risk of ovarian cancer begins to increase at age 35 for *BRCA1* and age 40 for *BRCA2* [21]*.* This has prompted modern guidelines to recommend BSO between ages 35–40 for *BRCA1* and 40–45 for *BRCA2* [22]*.* Despite this, rates of risk-reducing BSO by these age-specific targets remain low, with one international database study estimating a mean age of 45 years for *BRCA1* carriers and 48 years for *BRCA2* [23]*.* Our study mirrors these findings in that, while nearly one-third of clinic attendees were 40 years of age or less at the time of their appointment, only 30.6% of eligible women had a risk-reducing BSO before age 40 and the majority of these were in women with *BRCA2* pathogenic variants. We did, however, see increased OCP use over the study period, suggesting that while timing of risk-reducing BSO remains an area of focus for counselling and further exploration, adoption of less invasive risk-reduction strategies may be increasing.

The majority of women in this cohort felt that they had received adequate information from the HGRRC to be able to make an informed decision around risk reduction strategies. Patients perceived the greatest merit in the education delivered by geneticists and genetic counsellors, despite these care providers meeting with patients at a separate appointment outside of the HGRRC. With high attendance at the HGRRC, this suggests patients’ complex decisions making is better facilitated through gathering and synthesizing information at a few points along their care path before making their decision. While many institutions have moved to reflexive models of genetic testing where primary oncology providers facilitate testing directly, these findings suggest enduring value of a multidisciplinary approach and retention of this expertise.

Recommendations around risk reduction strategies have evolved over time with new data availability. As our study was conducted over a 10-year period, it is plausible the dissemination of this knowledge to patients differed over time and influenced uptake of risk-reducing options over the study period. Additionally, outcome data of self-reported questionnaires is susceptible to response bias. We aimed to mitigate this by offering multiple modalities of response and examining characteristics of respondents and non-respondents to characterize these groups. The characteristics of questionnaire respondents were similar except in risk-reducing BSO status. This suggests that although the response rate to the survey was only 50%, it is likely to reflect the study cohort.

Strengths of this study include the provincial catchment area, detailed information around timing of diagnoses and treatment derived from established databases, integration of clinical information from both the genetics and gynaecologic oncology service, and evaluation of patient perspective of this clinic. The population in Nova Scotia is relatively homogeneous [24], and it is therefore unsurprising that most patients in this study were of Western European origin. This may limit the generalizability of these findings to more diverse settings.

## Conclusion

The majority of Nova Scotian women with HBOC are referred to and counselled through HGRRC. Participants identified information from genetic counsellors as most valuable in risk-reduction decision-making, highlighting the importance of a multidisciplinary team. The majority of women seen in HGRRC chose to have risk-reducing gynaecologic surgery, which suggests that our care model supports informed choice within this population.

## Supplementary Information

**Additional file 1.** Patient Questionnaire.

## Data Availability

The datasets analysed during the current study are not publicly available due to NSHA REB restrictions. Please contact the corresponding author for further information related to this dataset.

## Abbreviations

BSO: Bilateral salpingo-oophorectomy
BRCA: Breast cancer gene
HBOC: Hereditary breast and ovarian cancer
HGRRC: Hereditary Gynaecologic Risk Reduction Clinic
OCP: Oral contraceptive pill

## References

[1] Mavaddat N, Peock S, Frost D, Ellis S, Platte R, Fineberg E (2013). Cancer risks for BRCA1 and BRCA2 mutation carriers: results from prospective analysis of EMBRACE. J Natl Cancer Inst.

[2] Buys SS, Partridge E, Black A, Johnson CC, Lamerato L, Isaacs C (2011). Effect of screening on ovarian cancer mortality: the prostate, lung, colorectal and ovarian (plco) cancer screening randomized controlled trial. JAMA.

[3] Jacobs IJ, Menon U, Ryan A, Gentry-Maharaj A, Burnell M, Kalsi JK (2016). Ovarian cancer screening and mortality in the UK collaborative trial of ovarian cancer screening (UKCTOCS): a randomised controlled trial. Lancet.

[4] Iodice S, Barile M, Rotmensz N, Feroce I, Bonanni B, Radice P (2010). Oral contraceptive use and breast or ovarian cancer risk in BRCA1/2 carriers: a meta-analysis. Eur J Cancer.

[5] Finch AP, Lubinski J, Moller P, Singer CF, Karlan B, Senter L (2014). Impact of oophorectomy on cancer incidence and mortality in women with a BRCA1 or BRCA2 mutation. J Clin Oncol.

[6] Giannakeas V, Sopik V, Shestopaloff K, Iqbal J, Rosen B, Akbari M (2015). A model for estimating ovarian cancer risk: application for preventive oophorectomy. Gynecol Oncol.

[7] Horsman D, Wilson BJ, Avard D, Meschino WS, Kim Sing C, Plante M (2007). Clinical management recommendations for surveillance and risk-reduction strategies for hereditary breast and ovarian cancer among individuals carrying a deleterious BRCA1 or BRCA2 mutation. J Obstet Gynaecol Can.

[8] Rebbeck TR, Kauff ND, Domchek SM (2009). Meta-analysis of risk reduction estimates associated with risk-reducing salpingo-oophorectomy in BRCA1 or BRCA2 mutation carriers. J Natl Cancer Inst.

[9] Kurian AW, Munoz DF, Rust P, Schackmann EA, Smith M, Clarke L (2012). Online tool to guide decisions for BRCA1/2 mutation carriers. J Clin Oncol.

[10] Owens DK, Davidson KW, Krist AH, Barry MJ, Cabana M, Caughey AB (2019). Risk assessment, genetic counseling, and genetic testing for BRCA-related cancer: US preventive services task force recommendation statement. JAMA.

[11] Walker JL, Powell CB, Chen L, Carter J, Bae J, Victoria L (2015). Society of gynecologic oncology recommendations for the prevention of ovarian cancer. Cancer.

[12] Engel NJ, Gordon P, Thull DL, Dudley B, Herstine J, Jankowitz RC (2012). A multidisciplinary clinic for individualizing management of patients at increased risk for breast and gynecologic cancer. Fam Cancer.

[13] Bancroft EK, Locke I, Ardern-Jones A, D'Mello L, McReynolds K, Lennard F (2010). The carrier clinic: an evaluation of a novel clinic dedicated to the follow-up of brca1 and brca2 carriers-implications for oncogenetics practice. J Med Genet.

[14] Yerushalmi R, Rizel S, Zoref D (2016). A dedicated follow-up clinic for BRCA mutation carriers. Isr Med Assoc J.

[15] Canadian Cancer Statistics Advisory Committee in collaboration with the Canadian Cancer Society, Statistics Canada and the Public Health Agency of Canada. Canadian Cancer Statistics 2021. Toronto: Canadian Cancer Society; 2021. http://www.cancer.ca/Canadian-Cancer-Statistics-2021-EN. Accessed 20 Apr 2022.

[16] Harris PA, Taylor R, Minor BL, Elliott V, Fernandez M, O'Neal L (2019). The REDCap consortium: building an international community of software platform partners. J Biomed Inform.

[17] Harris PA, Taylor R, Thielke R, Payne J, Gonzalez N, Conde JG (2009). Research electronic data capture (REDCap)—a metadata-driven methodology and workflow process for providing translational research informatics support. J Biomed Inform.

[18] Beattie MS, Crawford B, Lin F, Vittinghoff E, Ziegler J (2009). Uptake, time course, and predictors of risk-reducing surgeries in BRCA carriers. Genet Test Mole Biomark.

[19] Miller S, Roussi P, Daly M, Scarpato J (2010). New strategies in ovarian cancer: uptake and experience of women at high risk of ovarian cancer who are considering risk-reducing salpingo-oophorectomy. Clin Cancer Res.

[20] Metcalfe K, Eisen A, Senter L, Armel S, Bordeleau L, Meschino WS (2019). International trends in the uptake of cancer risk reduction strategies in women with a BRCA1 or BRCA2 mutation. Br J Cancer.

[21] Kotsopoulos J, Gronwald J, Karlan B, Rosen B, Huzarski T, Moller P (2018). Age-specific ovarian cancer risks among women with a BRCA1 or BRCA2 mutation. Gynecol Oncol.

[22] National Comprehensive Cancer Network (2021). Genetic/familial high-risk assessment: breast, ovarian and pancreatic.

[23] Rebbeck TR, Mitra N, Wan F, Sinilnikova OM, Healey S, McGuffog L (2015). Association of type and location of BRCA1 and BRCA2 mutations with risk of breast and ovarian cancer. JAMA.

[24] Statistics Canada. Nova Scotia and Canada. Census profile. 2016 Census. Statistics Canada Catalogue no. 98-316-X2016001. Ottawa. Released November 29, 2017.

