# Supplementary material for: Hereditary ovarian cancer risk reduction: a retrospective evaluation of patient perspectives and service provision at a regional hereditary gynaecologic cancer clinic 2006–2016
Source: BMC Womens Health. 2022 Jun 29;22:263. doi: 10.1186/s12905-022-01844-5 (PMC9245201; doi:10.1186/s12905-022-01844-5)
Supplement: Supplementary file 1 — Additional file 1. Patient Questionnaire. [file 12905_2022_1844_MOESM1_ESM.pdf]

BRCA Risk Reduction Survey

Please complete the survey below.

Thank you!

Please enter your study ID number:	__________________________
When you were seen for the results of your genetic testing, did you understand that women with a BRCA gene mutation have an increased risk for ovarian cancer compared to other women in Canada?	◯ Yes<br>◯ No<br>◯ I don't know
Why did you choose to have genetic testing?	◯ Diagnosis of cancer<br>◯ Family history<br>◯ Other
Please explain:	__________________________
Were you offered an appointment at the Dickson Centre Gynaecology Clinic to talk about what can be done to decrease the risk of ovarian cancer?	◯ Yes<br>◯ No<br>◯ I don't know
Did you see another local gynaecologist after your appointment at the Dickson Centre Gynaecology Clinic?	◯ Yes<br>◯ No<br>◯ I don't know
Was it only to discuss ovarian cancer risk-reduction?	◯ Yes<br>◯ No
Had you seen this gynaecologist previously?	◯ Yes<br>◯ No
Did you still need further information from your local gynaecologist about ovarian cancer risk-reduction?	◯ Yes<br>◯ No
If you were not referred to the Dickson Centre Gynaecology Clinic, was it because:	◯ Previous surgery to remove ovaries or fallopian tubes<br>◯ General health<br>◯ Other
Please explain:	__________________________
Did you request to see someone in your own community?	◯ Yes<br>◯ No
Where did you see them?	__________________________

KOFAX

Do you have a close relative who has had ovarian cancer?	◯ Yes<br>◯ No<br>◯ I don't know
Did they influence your decision to have an appointment at the Dickson Centre Gynaecology Clinic?	◯ Yes<br>◯ No<br>◯ I don't know
Do you feel that you had been given enough information to make the right decision about ovarian cancer risk-reduction for you?	◯ Yes<br>◯ No<br>◯ I don't know

50% Complete

.

Have you ever taken the birth control pill?	◯ Yes<br>◯ No<br>◯ I don't know
For how many years did you take the birth control pill?	__________________________________
Is one of the reasons you took the birth control pill for ovarian cancer risk-reduction?	◯ Yes<br>◯ No<br>◯ I don't know<br>◯ I prefer not to answer
Have you had your tubes and/or ovaries removed?	◯ Yes<br>◯ No<br>◯ I don't know<br>◯ I prefer not to answer
What year (YYYY) did you have your tubes and/or ovaries removed?	__________________________________
At what hospital did you have your tubes and/or ovaries removed?	__________________________________
Have you used any of the following to get information on ovarian cancer?	☐ Internet<br>☐ Pamphlets<br>☐ Books<br>☐ Family members or friends<br>☐ Other
Other - please explain:	__________________________________

KOFAX

Which services did you find helpful? Please select an answer for each item.

	Yes - helpful	No - not helpful
Genetics Clinic	◯	◯
Genetic Counsellor	◯	◯
Local Gynaecologist	◯	◯
Dickson Centre Gynaecology Clinic	◯	◯
Family Doctor/Nurse Practitioner	◯	◯
Ovarian Cancer Canada	◯	◯
Other	◯	◯

Other - please describe: __________________________________

What other information about decreasing your risk of ovarian cancer would have been helpful for you to know? __________________________________

Why? __________________________________

Have you ever been diagnosed with breast cancer?
◯ Yes
◯ No

Have you had an MRI and/or mammogram in the last 1-2 years to screen for breast cancer?
◯ Yes
◯ No
◯ I don't know

Have you had a bilateral mastectomy?
◯ Yes
◯ No

If you did not have an opportunity to discuss your risk of ovarian cancer and what the options are to decrease your risk, would you like an appointment at the Dickson Centre Gynaecology Clinic to do so?
◯ Yes
◯ No

Would you be interested in being contacted for a future study about hereditary cancer?
◯ Yes
◯ No

If there are questions regarding your questionnaire answers, may we contact you again?
◯ Yes
◯ No

As a token of our appreciation, are you interested in entering your name into a draw for one of ten $50 gift cards (Sobeys, Indigo/Chapters, Shoppers Drug Mart)?
◯ Yes
◯ No

Full name: __________________________________

KOFAX

Date of birth (DD/MM/YY): __________________________________

Phone number: ______________________________

Email: ______________________________

Are you interested in receiving a copy of the survey results?
◯ Yes
◯ No

Thank you for completing this survey!

If you indicated that you wished to receive a copy of the survey results, these will be mailed to you once this project is complete.

If you have questions or need help with the questionnaire, please contact us:

Email: janet.slaunwhite@iwk.nshealth.ca
Phone: (902) 470-6464

KOFAX
